# *Streptococcus equi* Subspecies *zooepidemicus* and Sudden Deaths in Swine, Canada 

**DOI:** 10.3201/eid2610.191485

**Published:** 2020-10

**Authors:** Matheus de O. Costa, Brad Lage

**Affiliations:** University of Minnesota, St. Paul, Minnesota, USA (M.O. Costa);; University of Saskatchewan, Saskatoon, Saskatchewan, Canada (M.O. Costa);; Utrecht University, Utrecht, the Netherlands (M.O. Costa);; Maple Leaf Agri-Farms, Landmark, Manitoba, Canada (B. Lage)

**Keywords:** Antimicrobial resistance, bacteria, Canada, cocci, lymph nodes, mortality, Mycoplasma hyopneumoniae, outbreak, pneumonia, sows, Streptococcus equi, sudden deaths, swine, zooepidemicus

## Abstract

Historically described as a commensal of the swine upper respiratory tract, *Streptococcus equi* subspecies *zooepidemicus* was previously reported as an important swine pathogen only in Asia. Here we report the isolation and whole genome characterization of *S. equi* subsp. *zooepidemicus* associated with a sudden death outbreak in pigs in Canada.

*Streptococcus equi* subspecies *zooepidemicus* is considered a commensal and opportunistic pathogen of several warm-blooded hosts, including humans, horses, canines, and swine. It is a gram-positive, β-hemolytic coccus belonging to the Lancefield group C and can cause severe disease characterized by pneumonia, septicemia, and meningitis (*1*,*2*). *S. equi* subsp. *zooepidemicus* has been suggested as a normal inhabitant of the palatine tonsils of pigs, being detected by both culture and high-throughput sequencing in samples collected from healthy animals (*3*). However, strains virulent to pigs have also been reported, particularly associated with high-mortality outbreaks of sudden death and respiratory disease in China (*4*). No vaccines are available for this pathogen, and control and prevention methods are rarely applied because of its normally harmless commensal nature in swine. Here, we report an outbreak of sudden death associated with *S. equi* subsp. *zooepidemicus* in pigs housed in intensive commercial rearing facilities in Canada. 

In April 2019, an outbreak of sudden deaths and abortions occurred in 4 loose-housed, commercial sow farms (»9,000 sows) in a large vertically integrated swine system in Manitoba, Canada. This outbreak increased the cumulative death in the 3 affected sow herds by >1,000 animals over a 12-week period. The abortion rate during this time was »11× normal. 

The sows were often described as apparently healthy during morning checks. However, over the course of hours, infected sows would become unwilling to stand, develop fever and lethargy, then die with no other apparent clinical signs. Other sows would abort and then go on to develop similar symptoms. Stress factors, such as mixing groups of sows and the presence of other sick animals, appeared to exacerbate outbreaks within pens. 

Animals were electronically fed a commercial grade, nutritionally balanced diet and had ad libitum access to water. Gross postmortem examination of multiple animals, either euthanized or recently deceased, revealed rhinitis (mild, diffuse mucopurulent discharge); pulmonary edema; gall bladder edema; and hemorrhagic lymphadenopathy (tan-colored to hemorrhagic), consisting of submandibular, cervical neck, and bronchial lymph nodes. These signs, taken together, suggest sepsis. We used real-time PCR to test all of the dead sows for porcine reproductive and respiratory syndrome virus, *Mycoplasma hyopneumoniae*, simian immunodeficiency virus A, and porcine circovirus types 2 and 3; results were negative for each. 

In parallel, we observed gram-positive cocci in imprints from heart and submandibular lymph nodes. After aerobic bacterial culture followed by matrix-assisted laser desorption/ionization-time of flight (MALDI-TOF) mass spectrometry for identification of isolates revealed varying levels of *S. equi* subsp. *zooepidemicus* in liver, kidney, heart, brain, lung, spleen, and submandibular lymph nodes. Isolate identification was confirmed by 2 different veterinary diagnostic laboratories. We found isolates SAMN13058951, SAMN13058952, SAMN13058953, SAMN13058954, SAMN13058955, SAMN13058956, and SAMN13058957 resistant to lincomycin, neomycin, and tetracycline and susceptible to ampicillin, ceftiofur, penicillin, and tilmicosin in a Kirby-Bauer disk diffusion assay. 

We extracted DNA from isolates using DNeasy Powersoil Pro kit (QIAGEN, https://www.qiagen.com), quantified by Nanodrop (3300) and PicoGreen (Quant-iT dsDNA; Invitrogen, https://www.thermofisher.com), then processed it for sequencing using a Illumina Nextera XT library prep kit (Illumina, https://www.illumina.com). We performed sequencing using MiSeq Nano V2, 2×250 paired-end (Illumina). Samples yielded an average of 149,017 high quality reads, suggesting 50× coverage. We conducted genome assembly, annotation, and downstream analyses using the PATRIC package (*5*). Genomes averaged 2.1 million bp in size and 41.34% in guanine-cytosine content. 

All isolates were similar to previously published *S. equi* subsp. *zooepidemicus* genomes (Figure), demonstrating a whole-genome average nucleotide identity score of 99.7% to strain ATCC35246. This particular strain was isolated from a septicemic pig during an outbreak that killed >300,000 pigs in Sichuan Province, China, in 1976 (*6*). All isolates had an average nucleotide identity score of 97.3% compared with *S. equi* subsp. *equi* strain 4047, an isolate considered virulent and obtained from a horse diagnosed with strangles in the United Kingdom (*7*). In addition, all isolates obtained from pigs, regardless of from which outbreak, were profiled as multilocus sequence type 194, including strains ATCC35246 and CY (also recovered from a diseased pig in China) (*8*). Antimicrobial resistance genes identified in isolates from this outbreak included *gidB*, *S12p* (streptomycin-resistant), *rpoB* (rifampin-resistant), *S10p* (tetracycline-resistant), *kasA* (triclosan-resistant), *PgsA*, *LiaR*, *LiaS* (daptomycin-resistant), *folA*, *Dft* (trimethoprim-resistant), *folP* (sulfadiazine-resistant), and *FabK* (triclosan-resistant). Virulence factors found, including the previously described *szm*, *szp*, *lmb*, *fbpZ*, *skc*, and *has* operons and *mga* regulon (*9*), help explain the high virulence of these isolates. 

**Figure:**
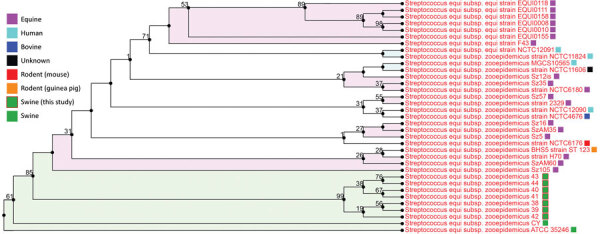
Phylogenetic tree (all-shared proteins) of *Streptococcus equi* subspecies *zooepidemicus* whole-genome sequences obtained from outbreak in pigs from Canada (blue blocks, PRJNA578379), compared with previously characterized genome sequences from GenBank (n = 28). Tree inferred using BLAST (https://blast.ncbi.nlm.nih.gov), followed by FastTree within the PATRIC package (*5*). Support values shown indicate the number of times a particular branch was observed in the support trees using gene-wise jackknifing. Shaded colors reflect similar host taxonomy associated with a branch (>3 isolates).

Taken together, these findings suggest the emergence of *S. equi* subsp. *zooepidemicus* sequence type 194 as a cause of death in pigs in Canada and possibly other regions of North America. This specific sequence type seems to be particularly virulent to pigs, for reasons that remain unexplained. Given the clinical presentation described here, this pathogen requires special attention and should no longer be overlooked when conducting diagnostic investigations, despite its historically accepted status as a commensal organism. 
